# Evaluating clinical outcomes of routinely delivered task-shared care for depression in rural Haiti

**DOI:** 10.1017/gmh.2021.17

**Published:** 2021-05-31

**Authors:** Alexandra L. Rose, Ryan McBain, Jesse Wilson, Sarah F. Coleman, Emmanuel Mathieu, J. Reginald Fils-Aimé, Emmeline Affricot, Tatiana Thérosmé, Wilder Dubuisson, Eddy Eustache, Stephanie L. Smith, Giuseppe Raviola

**Affiliations:** 1 Department of Psychology, University of Maryland, College Park, USA; 2 Partners in Health, Boston, USA; 3 RAND Corporation, Boston, USA; 4 Zanmi Lasante, Mirebalais, Haiti; 5 Department of Global Health and Social Medicine, Harvard Medical School, Boston, USA

**Keywords:** Global mental health delivery, Haiti, major depressive disorder, psychotherapy, task sharing

## Abstract

**Background:**

There is a growing literature in support of the effectiveness of task-shared mental health interventions in resource-limited settings globally. However, despite evidence that effect sizes are greater in research studies than actual care, the literature is sparse on the impact of such interventions as delivered in routine care. In this paper, we examine the clinical outcomes of routine depression care in a task-shared mental health system established in rural Haiti by the international health care organization Partners In Health, in collaboration with the Haitian Ministry of Health, following the 2010 earthquake.

**Methods:**

For patients seeking depression care betw|een January 2016 and December 2019, we conducted mixed-effects longitudinal regression to quantify the effect of depression visit dose on symptoms, incorporating interaction effects to examine the relationship between baseline severity and dose.

**Results:**

306 patients attended 2052 visits. Each visit was associated with an average reduction of 1.11 in depression score (range 0–39), controlling for sex, age, and days in treatment (95% CI −1.478 to −0.91; *p <* 0.001). Patients with more severe symptoms experienced greater improvement as a function of visits (*p* = 0.04). Psychotherapy was provided less frequently and medication more often than expected for patients with moderate symptoms.

**Conclusions:**

Our findings support the potential positive impact of scaling up routine mental health services in low- and middle-income countries, despite greater than expected variability in service provision, as well as the importance of understanding potential barriers and facilitators to care as they occur in resource-limited settings.

## Background

Throughout the world, the majority of persons with mental and behavioral disorders live without access to mental health treatment. In low- and middle-income countries (LMICs), almost half of people needing treatment do not access care at all (Thornicroft *et al*., 2017). Among those who access care, access to minimally adequate care is limited, with only an estimated 3.7% of people accessing depression treatment in LMICs accessing minimally adequate care. This treatment gap exists in part because the vast majority of people affected by mental disorders live in LMICs, where resources for mental health services have traditionally been limited and highly centralized (Jacob and Patel, 2014). In recent years, researchers and implementers have made considerable progress developing and testing evidence-based interventions to detect and treat mental health conditions in low- and middle-income settings, particularly in the care of common mental disorders such as depression (Singla *et al*., 2017). This has included approaches such as ‘task-sharing’ of care by non-specialists through the mobilization of peers and community health workers (CHWs), as well as adaptation of evidence-based psychotherapies such as cognitive-behavioral therapy and interpersonal therapy for delivery in low-resource settings (Legha *et al*., 2015; Verdeli *et al*., 2016; Hoeft *et al*., 2018). Despite the generation of rich evidence on clinically effective task-shared interventions for common mental disorders in low- and middle-income settings in recent years, the actual implementation and evaluation of such services in routine care in low-resource health systems remains limited. Notably, the literature on the delivery and clinical outcomes of evidence-based interventions as routinely delivered in LMICs is sparse even for the priority disorder of major depression, which was the 13th leading cause of disability-adjusted-life-years across all age groups worldwide in 2019 and ranks even higher among young people (Vos *et al*., 2020).

Evaluation of the delivery and clinical outcomes of task-shared mental health interventions in routine care is critical as there is evidence of overestimation of effect sizes in psychological research and smaller effect sizes when deploying psychological interventions in actual practice than in research environments (Weisz *et al*., 1995; Cuijpers *et al*., 2020a). Studies examining the delivery and outcomes of depression care as it occurs in routine care in resource-limited settings could help inform estimates of the potential impact of scale up of task-shared mental health interventions, while also elucidating further implementation challenges (Murray *et al*., 2014). Zanmi Lasante (ZL) is a local health care delivery organization founded in Haiti in 1987 to provide health care services to the poorest people in Haiti, managing its work in partnership with the Haitian Ministry of Health (Ministère de la Santé Publique et de la Population (MSPP)) in a catchment area that currently serves 1.5 million people. Its sister organization, Partners In Health (PIH), provides human and technical resources through partnerships with global academic institutions. An initial qualitative context assessment completed by ZL following the 2010 earthquake identified that formal depression care should be a major priority for new mental health service development (Interuniversity Institute for Research and Development, 2011). Over the 9 years since the MSPP, ZL and PIH have worked together to develop and implement a sustained model of community-based mental health care in Haiti, grounded in depression care, with care delivered via structured care pathways by CHWs in the community and psychologists, social workers, and physicians at facilities (Raviola *et al*., 2020).

This paper leverages routinely collected data from January 2016 to December 2019 to examine the clinical outcomes of the ZL intervention package for depression, making it among the first studies examining outcomes of routine task-shared mental health care in a LMIC. We hypothesized that, controlling for duration of time enrolled in care, patients with a diagnosis of depression would experience a reduction of depression symptoms as a function of the number of treatment visits they attended. We also expected that in alignment with ZL clinical care pathways, psychotherapy would be most frequently provided to patients with moderate depression, and medication would be most frequently provided to patients with severe depression.

## Methods

### Setting

The Haiti earthquake of January 12, 2010 resulted in an estimated 222000 people killed, 300500 injured, and more than 1.5 million people homeless (United Nations Office for the Coordination of Humanitarian Affairs, 2011). Both major depressive disorder and anxiety had previously been listed within the top five leading causes of years lived with disability in Haiti, and were expected to increase in prevalence following this humanitarian disaster (GBD Profile: Haiti, 2010; DesRoches *et al*., 2011).

**Fig. 1.**
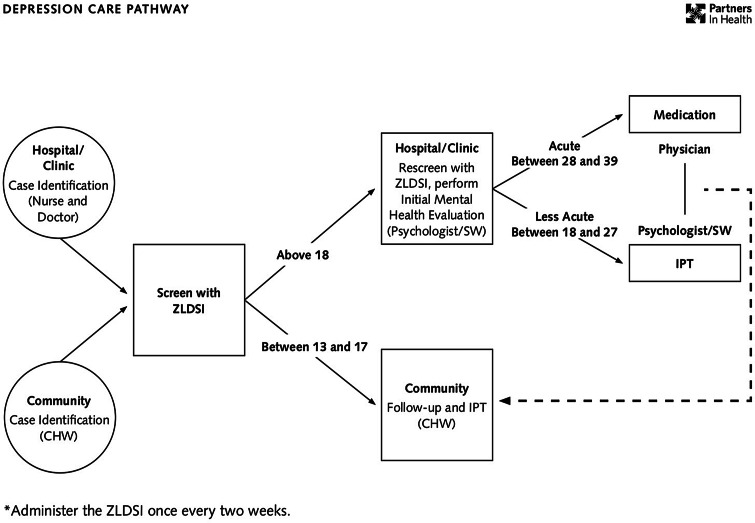
Zanmi Lasante depression care pathway.

The stepped care pathway for depression care at ZL, formally implemented in 2013 after a period of emergency response and curriculum development, is presented in Fig. 1. In brief, patients are screened by CHWs in the community, or nurses and physicians at the facility level, using a locally developed and validated depression scale (Rasmussen *et al*., 2015). Patients with mild depression symptoms are primarily referred to a community health worker for psychoeducation and possibly for psychotherapy and are primarily seen in the community. Patients with moderate or severe symptoms of depression are rescreened by a second facility provider and referred to a psychologist or social worker for a full mental health evaluation. Patients with moderate symptoms see a psychologist or social worker for psychoeducation and psychotherapy at health facilities. Patients with more severe symptoms see a physician for medication at health facilities and, as needed, a CHW for psychoeducation/psychotherapy and follow-up in the community. Between January 2016 and December 2019, a total of 30486 mental health care visits across a range of mental disorders were documented at ZL facilities.

### Clinical assessment

Paper forms are used at all community and health facility visits for a mental health condition to document clinical care. Versions of these forms can be accessed in English online (Partners in Health Curriculum Toolkit, 2016). Key data for all patients at health facilities are entered into an anonymized electronic data collection system following each visit, a database which was used for this study. CHW interventions are not yet represented in this database.

### Measures

The Zanmi Lasante Depression Symptom Inventory (ZLDSI) represents the primary clinical measure for depression care at ZL. The ZLDSI is a 13-item tool created and validated in the Haitian context and administered in Haitian Kreyol (Rasmussen *et al*., 2015). It asks about depression symptoms over the previous 2-week period, including difficulty sleeping, reduced appetite, reduced motivation, and suicidal ideation. It can be used both as an initial screener for depression and to track depression symptoms over time. It is therefore meant to be recorded at all initial visits with new patients and at all follow-up visits with patients with depression symptoms. Response scores range from 0 to 39, with scores 0–12 indicating sub-threshold depressive symptoms, 13–17 indicating mild depression, 18–27 indicating moderate depression, and 28–39 indicating severe depression.

We also extracted two additional measure sets for this analysis. First, we quantified the number of clinical visits at which individuals received any mental health care – inclusive of psychotherapeutic interventions (e.g. interpersonal therapy, relaxation) and antidepressant therapy. Second, we recorded patient sex and age at the time of enrollment in depression care and location of service provision.

### Inclusion criteria

From the entire set of mental health visits recorded in the electronic data collection system between January 1, 2016, and December 31, 2019, patients were included in this analysis if they met the following inclusion criteria: (1) Depression listed as the most frequent primary diagnosis over the course of visits, as defined by the provider at the time of care; (2) at least two visits recorded over a minimum of 30 days; (3) at least two ZLDSI scores recorded over the course of visits; and (4) A ZLDSI of at least 13 at baseline, or the threshold for mild depression. Patients were excluded if they had been prescribed medication not indicated for treatment of depression. After finalizing the sample, patients were categorized into baseline severity categories using the ZLDSI score at the first visit. We excluded patients that had only attended services once or who did not have two ZLDSI scores, as it would be possible to examine change in symptomology over time without imputation. We excluded patients who had attended two visits in under 30 days as this would have fallen under the recommended 4 weeks for minimally adequate pharmacotherapy treatment (Wang *et al*., 2007; Thornicroft *et al*., 2017).

### Statistical analysis

Descriptive statistics were calculated for the resulting sample, with mean and standard deviation reported for continuous variables and frequency and percentages reported for categorical variables. We compared groups based on severity category (mild v. moderate v. severe) using *χ*^2^ tests and one-way ANOVAs. Based on available resources, the study was powered to detect a standardized effect size (Cohen's *δ*) of *δ* = 0.40 when examining the effect of treatment (behavioral therapy and antidepressant therapy), assuming a serial correlation of *r* = 0.50 and a target sample size of *n* = 176 enrollees. With the analytic sample size of *n* = 306, statistical power exceeded 0.90.

We used mixed-effects longitudinal regression analysis to examine the relationship between level of treatment exposure (measured as number of clinical visits) and magnitude of change in ZLDSI score from enrollment to endline, defined as the last date for which patients were recorded as receiving care. In the absence of a comparison group, this constitutes a longitudinal extension of pre-post treatment analysis that is common in the empirical literature (Adams *et al*., 2015; Bernstein *et al*., 2016; Wagenaar *et al*., 2020). Due to the use of routinely collected data for this study and based on the observed efficacy of antidepressant and behavioral therapy, the research team deemed that a comparison group deprived of treatment would not be acceptable within the health system and would be considered to lack equipoise.

In addition to number of visits, we included number of days in care as a fixed effect – to account for potential ‘regression to the mean’ whereby patients may improve in terms of their symptom severity merely by the passing of time, irrespective of treatment. Accounting for regression to the mean is necessary in a pre-post intervention study design (Flett *et al*., 1995; Linden, 2013; Hengartner, 2020). We also included patient age and sex as additional covariates. Lastly, we included fixed effects for treatment facilities at which patients received care to account for possible variations in quality or frequency of treatment at the facility level. A random effect was included to account for autocorrelation – i.e. nesting of observations over time within patients. Ultimately, this model specification allowed us to determine whether greater treatment exposure (number of clinical visits) was associated with more significant depression symptom reduction, after adjusting for the role of regression to the mean (i.e. time) and other covariates.

Potential departures from linearity were examined by examining normality of residuals in our analysis, including kernel density plots and standardized normal probability plots. No significant departures were observed. We identified one studentized residual that represented an outlier (>2 standard deviations from the mean). When removed, results were substantively unchanged and the Shapiro−Wilk W test for normality was not statistically significant. As such, we report results based on the full sample.

For secondary analyses, we incorporated a set of interactions effects. First, we examined the interaction between treatment dose and baseline symptom severity: looking at the comparative effects of clinical visits among patients classified as either mild, moderate or severely depressed prior to care. Regression analyses were conducted in STATA version 16.0 s.e. using the xtreg command, which accounts for missingness using a full information maximum likelihood approach.

## Results

306 patients, representing 12 health facilities throughout the ZL network, met inclusion criteria for analysis (Table 1). A total of 456 patients were excluded from analyses due to receipt of only one clinical visit over the study period. Additionally, 292 patients received more than one clinical visit but received care for fewer than 30 days; and 136 patients who received two or more clinical visits over 30 or more days were excluded due to an absence of ZLDSI information. Of the 306 patients included, the minimum duration of care for individuals included in the analysis was 30 days, the maximum was 1427 days, and the mean was 294 days. Patients in the analytic sample attended 2052 visits during the period under study – an average of 6.6 visits (s.d.: 5.1) per patient, with no differences in the average total number of visits attended across severity categories (*p* = 0.7). The largest severity group represented was moderate depression (*n* = 147; ZLDSI: 18–27), followed by severe (*n* = 99; ZLDSI: 28–39), and mild (*n* = 60; ZLDSI: 13–17). The mean baseline ZLDSI for the sample was 23.7 (s.d.: 6.58), with mean of 14.92 (s.d.: 1.51) for mild depression, 22.0 (s.d.: 2.77) for moderate depression, and 31.58 (s.d.: 2.82) for severe depression. The majority (86.3%) of the sample was female, with no differences in sex across severity categories (*p* = 0.7). The average age was 37.5 (s.d.: 14.5), with no differences in age across severity categories (*p* = 0.054). Though there were no differences in total visits across severity categories, patients with severe symptoms had a higher percentage of missed follow-up visits (42.3%) than patients with mild (35.1%) or moderate symptoms (38.5%), though these differences were not statistically significant. Across the entire sample, the percentage of missed follow up appointments was 39.4%.

**Table 1.** Demographic, clinical, and service use characteristics of sample (*n* = 306)

	Patients with mild symptoms (*n* = 60)	Patients with moderate symptoms (*n* = 147)	Patients with severe symptoms (*n* = 99)	Overall depression sample (*n* = 306)	*p* value
Gender – *n* (%)					
Female	50 (83.3)	127 (86.4)	87 (87.9)	264 (86.3)	0.7
Male	10 (16.7)	20 (13.6)	12 (12.1)	42 (13.7)	
Mean age (s.d.)	41.4 (17.2)	37.6 (14.6)	35.2 (12.1)	37.5 (14.5)	0.054
Mean baseline ZLDSI (s.d.)	14.92 (1.51)	22 (2.77)	31.58 (2.82)	23.7 (6.58)	<0.001
Number of visits over period	377	974	701	2,052	–
Mean number of visits over period per patient (s.d.)	6.2 (4.2)	6.5 (5.0)	6.9 (5.7)	6.6 (5.1)	0.7
Number of follow-up visits scheduled within period	231	718	516	1,465	–
Attended follow-up visit with 7 days of scheduled visit – *n* (%)					
Yes	100 (64.9)	328 (61.5)	240 (57.7)	668 (60.6)	0.2
No	54 (35.1)	205 (38.5)	176 (42.3)	435 (39.4)	
Psychotherapy intervention provided at visits – *n* (%)					
Yes	203 (53.8)	456 (46.8)	308 (43.9)	967 (47.1)	0.007
No	174 (46.12)	518 (53.1)	393 (56.1)	1085 (52.9)	
Any medication for depression prescribed at visit – *n* (%)					
Yes	115 (30.5)	558 (57.3)	485 (69.2)	1158 (56.4)	<0.001
No	262 (69.5)	416 (42.7)	216 (30.8)	894 (43.6)	

### Association between exposure and outcome

**Fig. 2.**
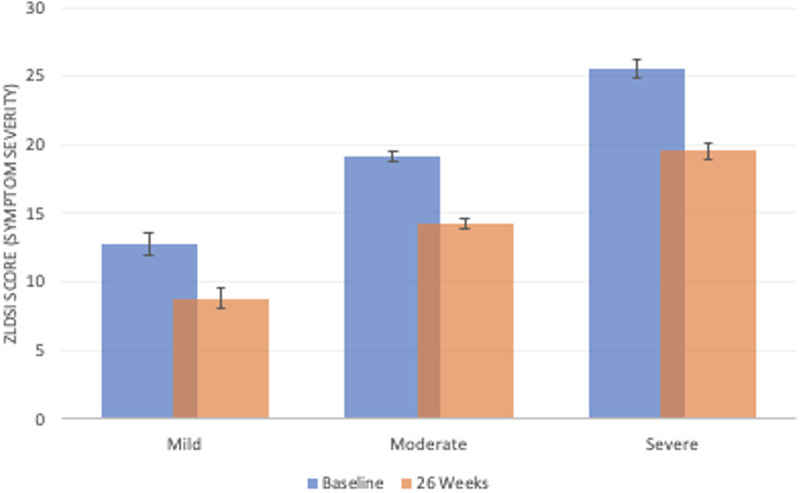
ZLDSI (depression severity) scores represent expected level of symptoms based on predictive margins from multivariable regression analyses. While ‘baseline’ represents the expected level of symptoms on Day 1, prior to receiving any depression care, ‘26 weeks’ represents the expected level of symptoms at the median duration for last depression care visit (26 weeks) and median number of depression care visits over this period (five visits). Error bars indicate standard errors around point estimates.

Examination of ZLDSI scores longitudinally reveals each depression treatment visit (medication and/or psychotherapy) recorded for a patient was associated with an average reduction in ZLDSI score, controlling for sex, age, and number of days in treatment (*β* **=** −1.107; *p* *<* 0.001; 95% CI −1.475 to −0.91), implying that our first hypothesis that greater exposure to treatment was associated with larger reductions in depression symptoms – adjusting for time – was supported. On average, a patient with a baseline ZLDSI score of 20 (moderate depression) would be expected after eight visits to have a ZLDSI score of around 10 (sub-threshold symptoms). The interaction model, which incorporated diagnostic symptom severity at baseline found significant variation in symptom reduction over time by severity classification (*β* *=* −0.20, *p:* 0.04; 95% CI: −0.40 to −0.005). Patients in the severe category experienced more symptom reduction than patients in the moderate category, and patients in the moderate category experienced more symptom reduction than patients in the mild category (Fig. 2).

### Services provided

Across the sample, psychotherapy was provided at 47.1% of visits. Provision of psychotherapy was significantly higher at visits for patients with mild symptoms than for patients with moderate or severe symptoms (*p* = 0.007), meaning our hypothesis that psychotherapy would be most frequently delivered to patients with moderate symptoms was not supported. Medications were prescribed at 56.4% of the visits and were significantly more common at visits for patients with severe symptoms than visits for patients with less severe symptoms (*p* < 0.001), meaning the hypothesis that medication would be more frequently delivered to those with severe symptoms was supported.

## Discussion

We find that an evidence-based model of depression care as delivered in public facilities in severely resource-limited rural Haiti demonstrates a significant relationship between a greater number of treatment visits and a reduction in depression symptoms. Though the reduction in ZLDSI score associated with one treatment visit was modest, the association is such that an eight-session course of treatment is associated with clinically meaningful reduction in symptoms similar to those seen in controlled trials (Cuijpers *et al*., 2020b). These findings are consistent with the larger global mental health literature that effective mental health care can be delivered by non-specialists (Hoeft *et al*., 2018) and also help provide critical confirmation that this care can be delivered without research infrastructure in a low-income country. These results highlight the promise of leveraging existing evidence to evaluate the clinical impact of mental health interventions as they are delivered in routine care and suggest a number of areas for future research.

Despite finding improvement across the sample, we found differences in outcomes based on the baseline severity of patients. This may be explained by more severely symptomatic patients having greater room for symptom improvement over the period (Linden, 2013). Notably, our findings exist in the context of more variation in intervention delivery than expected, which may have also had an impact on outcomes. Studies of community-based care in resource-limited contexts in the United States have also reported provider ‘drift’ from protocols even alongside effective outcomes (Marques *et al*., 2019). Understanding the drivers and impact of provider ‘drift’ outside of research settings remains an important area of work in mental health care delivery across contexts.

Psychotherapy was documented at less than half of visits for all patients in the sample and less often for patients with moderate depression than patients with mild depression. As psychotherapy is the primary intervention indicated for moderate depression by the care pathway at ZL and a central strategy for increasing access to mental health care globally (Bolton, 2019), it would be important to better understand the factors that contributed to these results. It is possible that in Haiti, where there exists a rich tradition of traditional practitioners providing care for mental health concerns, that some patients were choosing not to make use of psychotherapy services (Institute of Community and Family Psychiatry, 2010). Alternatively, in a health system in which mental health services were relatively new and demand was growing each year, it is possible that provider understanding of protocols was insufficient. Additionally, due to the small number of providers trained in psychotherapy at each health facility, it may not have been possible to provide psychotherapy to the number of clients with moderate symptoms with the time available. Though representing the smallest group in our sample, the number of patients with mild symptoms, who are meant to be treated primarily by CHWs outside of facilities, seen in this facility level data was also unexpected. Patients with mild symptoms attending facility-level services may have further limited time for psychotherapy service provision to patients with moderate symptoms. A better understanding of the staffing and the distribution of staffing necessary to adequately address the burden of illness is sorely needed within global health implementation more broadly (Mukherjee *et al*., 2019), and studies such as this serve to inform practical knowledge on mental health implementation.

Medication was most commonly provided to patients in the severe category, as expected. This is consistent with the ZL depression care pathway, which recommends medication provision only for severe cases, and highlights the importance of the medication supply chain in low-resource settings to support treatment for severe cases (Forum on Neuroscience and Nervous System Disorders *et al*., 2014). However, medication was also documented at over one third of visits categorized as mild depression visits and almost two thirds of visits categorized as moderate depression visits. It is possible that providers may have been overprescribing medication, mechanisms for which it would be important to better understand in future work. It would also be important to understand if and how under-provision of psychotherapy, as seen in patients with moderate depression in this sample, may in some cases relate to the overprovision of medication. Alternatively, it is possible providers may have been using other forms of assessment beyond the ZLDSI to assess clinical needs and inform treatment planning. This reflects the fact that screening tools, though essential in task-shared mental health care, are not sufficient in guiding clinical care. It also underscores the importance of sustained training and supervision in delivery of psychological interventions as well as psychopharmacology to ensure patient safety (Kemp *et al*., 2019).

Of note, the percentage of missed follow up appointments of 39.4% is not far above no-show rates for mental health services in high-income settings, which range from 27% to 31% in published studies (Gajwani, 2014; Clouse, Williams, and Harmon, 2015). One possible reason for this is that ZL facilities also provide primary care services, and patients may find it convenient to attend appointments when accessing other services as well. Another possible driver is the influence of CHWs linked to the facility-level services working in communities, prompting patients to attend visits. Overall, the relative similarity of missed appointments between this low resource-context and high-resource contexts is promising.

It is important to note that this is a relatively small analytic sample, which limits the generalizability of our findings. The small size of our sample likely stems in part from the relative newness and resource constraints of the mental health services at ZL even in 2016–2019. Despite the use of a locally developed and validated measure, it is also possible that depression was under-detected in our patient population on the whole. Recent literature across a number of LMIC settings has documented challenges in depression detection among task-shared providers (Rathod *et al*., 2018; Fekadu *et al*., 2020). We also included only patients treated primarily at facilities, an approach that excluded patients with mild symptoms treated in in the community. The use of routinely collected data, though the most feasible option in our context, also contributed to missing data, which further limited our sample size. In addition, decisions that we made to define our analyses excluded patients who only attended services once or briefly, who may have differed in important ways. It would be important to better understand any differences between patients who did not return or who attended services only briefly in future work, particularly due to the size of these groups. Further limitations include the inability to disentangle effects of pharmacotherapy from the effects of psychotherapy on patient outcomes or to control for quality of care delivered. Several of these limitations could be addressed in a future pragmatic trial focused on questions we have identified throughout this analysis. Additionally, clinical information about patients was not available prior to January 2016, though some patients may have been in care before then. We also did not examine providers' free text notes in the electronic data collection system, which may contain useful information about decisions made in treatment.

## Conclusions

We demonstrate a positive association between treatment visits and depression symptom reduction in routinely delivered task-shared services for depression care in a rural, low-resource health setting. Despite greater than expected variability in services provided, our findings are consistent with those of more controlled study designs and support the promise of global mental health scale up, while highlighting the need to continue understanding the impact and implementation challenges of task-shared mental health care as delivered in resource-limited settings.
